# Dissecting genetic diversity and genomic background of *Petunia* cultivars with contrasting growth habits

**DOI:** 10.1038/s41438-020-00373-2

**Published:** 2020-10-01

**Authors:** Yufang Guo, Ryan M. Warner

**Affiliations:** Department of Horticulture, Michigan State University, East Lansing, MI 48824 USA

**Keywords:** Plant genetics, Plant breeding

## Abstract

The cultivated petunia (*Petunia* ×*hybrida*) is derived from the progenitor species *P. axillaris* and *P. integrifolia*. The hybridization dates back only to the 1830s, though intensive breeding efforts have yielded cultivars exhibiting incredible diversity for many traits, including growth habit, flower color, and flower size. Until now, little is known about the genetic diversity and genomic background of modern cultivars. Here we selected a panel of 13 cultivars with contrasting growth habits and three wild species (the progenitors and *P. exserta*) to estimate the genomic contribution from the ancestral species and to study whether the variation of the genetic origin could be associated with different breeding programs or morphological variability. Transcriptome sequencing identified 1,164,566 SNPs representing 98.4% (32,451) of the transcripts that cover 99.2% (of 52,697,361 bp) of the *P. axillaris* transcriptome. Cultivars with an upright growth habit had more homozygous alleles and more *P. axillaris*-derived alleles than trailing cultivars, while mounded cultivars had intermediate heterozygosity. Unlike previous studies, we found the proportions of alleles derived from each progenitor species varied across cultivars but overall were not biased toward one progenitor species, suggesting diverse selection during cultivar development. For trailing cultivars, alleles potentially introgressed from other wild species (“out” alleles) were enriched. The “out” alleles were clustered in particular regions of chromosomes, suggesting that these regions may be hotspots of introgression. Transcripts in these regions were enriched with gene ontology terms associated with growth habit. This study provides novel insight into the contributions of progenitor species to the genomic background of modern petunia cultivars and identifies genome regions that may harbor genes conferring the trailing growth habit for further exploration.

## Introduction

The garden petunia, *Petunia* ×*hybrida*, consistently ranks among the top selling annual bedding plants in the U.S. and Europe. In 2018, petunia ranked first among annual bedding plants in the U.S., with a wholesale value of $141.7 million^1^. The genus *Petunia* contains 14 species found in Argentina, Bolivia, Brazil, and Uruguay^2^. Petunia is derived from crosses between *P. axillaris* and one or more species within the *P. integrifolia* clade^2–4^.

Domestication of the cultivated petunia is quite recent, with first mention of the hybrid occurring in 1835^3^. Since then, commercial breeders have released hundreds of cultivars exhibiting considerable diversity for traits like flower color, pattern and size, and growth habit^5^. The breeding history has sometimes involved hybridization with additional crop wild relative species. For example, petunia cultivars traditionally exhibited an upright growth habit^3^. However, a major new form of plant architecture for petunia, a strongly prostrate (trailing) and radial growth habit, was introduced with the release of the cultivar ‘Wave Purple’. This novel growth habit was likely the result of introgression of the species *P. altiplana* into commercial germplasm, as this is the only *Petunia* species exhibiting this growth habit^2^. Following the release of ‘Wave Purple’ and a second cultivar with a prostrate growth habit, petunia ‘Surfinia Red’, a large number of cultivars exhibiting growth habits intermediate to the upright and trailing types (mounded and mounding/trailing types) began to appear on the market.

In recent years, there has been a considerable increase in sequence availability for *Petunia* spp., including genome sequences for *P. axillaris* and *P. inflata* (a close relative of *P. integrifolia*)^5^, and transcriptome sequences for *P. axillaris*, *P. exserta*, *P*. ×*hybrida*, and *P. integrifolia*^6–8^. In addition, we have generated high-density single-nucleotide polymorphism (SNP)-based genetic maps for two interspecific *Petunia* recombinant inbred line (RIL) populations, including a *P. integrifolia* × *P. axillaris* population^9–12^. While these resources will greatly facilitate the elucidation of the genetic control of important traits, sequence resources representing the diversity of commercial petunia cultivars are currently lacking.

Bombarely et al.^5^ utilized the genome sequences of *P. axillaris* and the close *P. integrifolia* relative *P. inflata* and transcriptome data from three petunia laboratory strains, ‘Mitchell’, R27, and R143, to determine that the contribution of *P. axillaris* varied from 49.8 to 62.1%. However, these laboratory strains are not very representative of commercial cultivars, as R27 and R143 have been inbred for many generations, and ‘Mitchell’ is a doubled haploid derived from a cross between *P. axillaris* and petunia ‘Rose of Heaven’^13^. The contributions of *P. axillaris* and *P. integrifolia* to the genetic background of modern commercial cultivars are largely unknown.

The objectives of this study were to generate transcriptome-derived SNPs from a broad panel of petunia cultivars to evaluate the genetic diversity of petunia cultivars representing several breeding companies and a broad range of phenotypes, assess the relative contributions of *P. axillaris* and *P. integrifolia* to the genomic composition of these cultivars, identify genomic regions that may be derived from other (“outgroup”) crop wild relative species, and to further identify potential associations between prostrate growth habit and outgroup-derived genomic loci.

## Results

### SNP detection, annotation, and validation

Paired-end sequencing of three petunia cultivars (‘Wave Purple’, ‘Surfinia Red’, and ‘Madness Yellow’) contained 85–109 million high-quality reads. The average depth of coverage for uniquely mapped reads ranged from 37.1 to 53.3. The 10 other cultivars yielded 12.2 to 25.8 million high-quality single-end reads (Table 1). Transcriptome data for *P*. *axillaris*, *P*. *integrifolia*, and *P. exserta* were previously described^6^.

**Table 1:** Summary of the number of Illumina reads and the average depth of coverage for the petunia cultivar panel.

Cultivar	Tissue	Growth habit	Seq method	Raw reads (millions)	Unique reads (millions)	Uniquely mapped reads (millions)	Average depth of coverage	Percentage of bases with depth of coverage >10
Wave Purple	Flower and leaf	Trailing	Paired end	100.8	57.2	53.8 (94.5%)	53.3	51.8%
Surfinia Red	Flower and leaf	Trailing	Paired end	85.2	40.9	38.5 (94.4%)	37.1	44.7%
Madness Yellow	Flower and leaf	Upright	Paired end	109.1	66.6	62.8 (94.3%)	59.9	54.6%
Avalanche Salmon	Flower	Mounded	Single end	25.8	9	9.0 (100.0%)	5.4	19.3
Fantasy Rose	Flower	Upright	Single end	15.2	3.7	3.7 (100%)	2.2	5.7
Madness Red	Flower	Upright	Single end	16.5	6.4	6.4 (100.0%)	3.9	12.6
Orchid Daddy	Flower	Upright	Single end	17.1	4.3	4.3 (100%)	2.6	7.4
Storm Blue	Flower	Upright	Single end	15.2	5.7	5.7 (100%)	3.4	10.6
Super Cascade White	Flower	Upright	Single end	14.2	6.1	6.1 (100%)	3.7	11.8
Supertunia Yellow	Flower	Mounding/trailing	Single end	16.3	6.4	6.4 (100%)	3.8	12.4
Supertunia Vista Bubblegum	Flower	Mounding/trailing	Single end	24.1	8.4	8.4 (36.2%)	5.0	17.8
Supertunia Vista Silverberry	Flower	Mounding/trailing	Single end	12.2	5.2	5.2 (44.4%)	3.1	9.5
Tidal Wave Silver	flower	Mounding/trailing	Single end	15.4	6.2	6.2 (100%)	3.7	12.1

Cultivars were arranged according to (1) sequencing method, (2) growth habit, and (3) cultivar name.

A total of 1,353,141 SNPs were detected among the 16 genotypes (3 wild species and 13 cultivars). The SNPs were further filtered to exclude those where no polymorphisms were detected between the cultivars and the reference genotype (*P*. *axillaris*), which left 1,164,566 SNPs (entire dataset). These SNPs were located on 32,451 transcripts with a total length of 52,697,361 bp, which was 98.4% of the total transcripts and covered 99.2% of the *P*. *axillaris* transcriptome assembly length^6^. A total of 74,218 SNPs with no missing data (no missing) were detected among the 16 genotypes. This set of no missing SNPs were located on 7815 transcripts with a total length of 16,790,590 bp. They comprised 23.7% of the *P*. *axillaris* transcripts and covered 31.6% of the reported *P*. *axillaris* transcriptome^6^.

Within the entire SNP dataset, the SNP numbers varied greatly between cultivars (Table 2). For example, 61,959 SNPs were detected between ‘Vista Silverberry’ (average dp = 4.3) and ‘Orchid Daddy’ (average dp = 3.1), almost 3 times the number of SNPs between ‘Vista Silverberry’ and ‘Vista Bubblegum’ (23,040; average dp = 5). For cultivars, the number of SNPs was generally higher with *P*. *integrifolia* than with *P*. *axillaris* (Table 2). The number of SNPs between ‘Madness Yellow’ or ‘Fantasy Rose’ and *P*. *integrifolia* was almost twice that with *P*. *axillaris*. While for ‘Tidal Wave Silver’, ‘Vista Silverberry’, and ‘Vista Bubblegum’, similar number of SNPs were detected for the cultivars between both progenitor species (Table 2).

**Table 2:** Genome-wide comparison of transcriptome-derived SNP loci between 3 petunia wild species and 13 petunia cultivars.

	*P*. *axillaris*	*P*. *exserta*	*P. integrifolia*	Wave Purple	Surfinia Red	Madness Yellow	Avalanche Salmon	Fantasy Rose	Madness Red	Orchid Daddy	Storm Blue	Super Cascade White	Supertunia Yellow	Vista Bubblegum	Vista Silverberry
*P*. exserta*	253,078														
*P. integrifolia*	628,626	657,881													
Wave Purple	449,149	458,229	464,132												
Surfinia Red	409,935	419,543	407,033	136,348											
Madness Yellow	286,136	309,862	480,025	354,493	138,550										
Avalanche Salmon	150,191	161,811	231,798	163,132	161,710	143,316									
Fantasy Rose	48,090	54,151	92,009	66,440	73,289	46,631	43,055								
Madness Red	89,340	101,352	169,902	104,282	116,591	90,213	70,511	27,208							
Orchid Daddy	63,486	71,917	109,994	80,097	86,226	61,135	28,242	31,289	36,681						
Storm Blue	70,096	81,776	148,744	128,531	134,270	74,571	75,474	31,731	55,991	45,158					
Super Cascade White	75,714	86,551	165,554	139,880	146,067	59,649	81,135	34,038	56,936	41,727	44,032				
Supertunia Yellow	106,348	117,331	163,908	116,492	123,039	93,400	92,030	41,464	60,380	53,118	67,621	65,211			
Vista Bubblegum	194,955	201,353	194,538	157,134	151,848	166,059	127,682	67,831	112,454	74,676	108,910	107,729	104,643		
Vista Silverberry	123,311	128,082	124,183	101,359	99,439	106,608	90,951	57,705	85,294	61,959	84,748	82,172	80,331	23,040	
Tidal Wave Silver	139,493	145,887	141,848	123,227	121,287	116,508	113,650	62,835	95,959	71,252	86,267	81,579	84,877	86,579	67,807

Values indicate the number of SNPs for the pairwise comparison. For the species and cultivars ‘Wave Purple’, ‘Surfinia Red’, and ‘Madness Yellow’, 150 bp paired-end Illumina sequencing was employed. For the other cultivars, 50 bp single-end sequencing was used. Cultivars were arranged according to (1) sequencing method, (2) growth habit, and (3) cultivar name.

A total of 22 predicted SNPs were selected for validation. Sanger sequencing of the targeted amplified region showed that all of these predicted SNPs were detected. For the cultivars, 91.2% of these predicted SNPs were validated. The <9% mis-genotyped samples were mainly from heterozygous SNPs validated as homozygous. This normally happened for genotypes with low minor allele frequency. Overall, the SNP validation results showed high accuracy of the SNP prediction pipeline.

### Detecting regions under selection for petunia cultivars

The 74,218 SNP loci with no missing data were tested for signatures of selection based on their allelic differentiation from the expected neutrality^14^. The test was carried out by BayeScan, which incorporated the uncertainty on allele frequency and is thus suitable for datasets with small sample sizes^15^. When the cultivars were considered as one population and each wild species considered separately, BayeScan did not detect any outliers with default parameters at the default false discovery rate. When only cultivars were used, still no outliers were found, which indicated no significant signature of selection for petunia cultivars.

### The proportion of ancestral genomic contribution varies within petunia cultivars

**Fig. 1:**
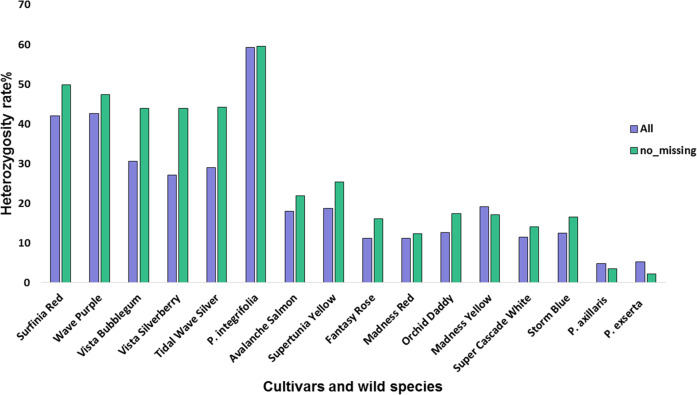
Heterozygosity level of the cultivars and the wild species using the transcriptome-derived SNPs, with the entire dataset (1,164,566 SNPs, represented by purple bars) and the SNPs with no missing data (74,218 SNPs, represented by green bars). The heterozygosity rate is calculated as the number of heterozygous SNPs divided by the total number of SNPs. The cultivars and wild species were arranged according to the PCA similarity on PC1 (Supplementary Fig. 1).

The transcriptome heterozygosity for each genotype was estimated using both the entire SNP dataset (1,164,566) and the no-missing SNP dataset (74,218) (Fig. 1). Both datasets showed that, among all 16 genotypes, *P. integrifolia* had the highest heterozygosity (59.3%) and *P*. *axillaris* and *P*. *exserta* had similar and the lowest heterozygosity. The cultivars had a range of heterozygosity rates sitting between the three wild species. In general, the trailing cultivars had higher transcriptome heterozygosity than the upright cultivars, and the mounded cultivars had heterozygosity rates in between. The low rates of transcriptome heterozygosity for the upright types (generally 10–15%) are particularly surprising, as most of these cultivars are F_1_ hybrids.

**Fig. 2:**
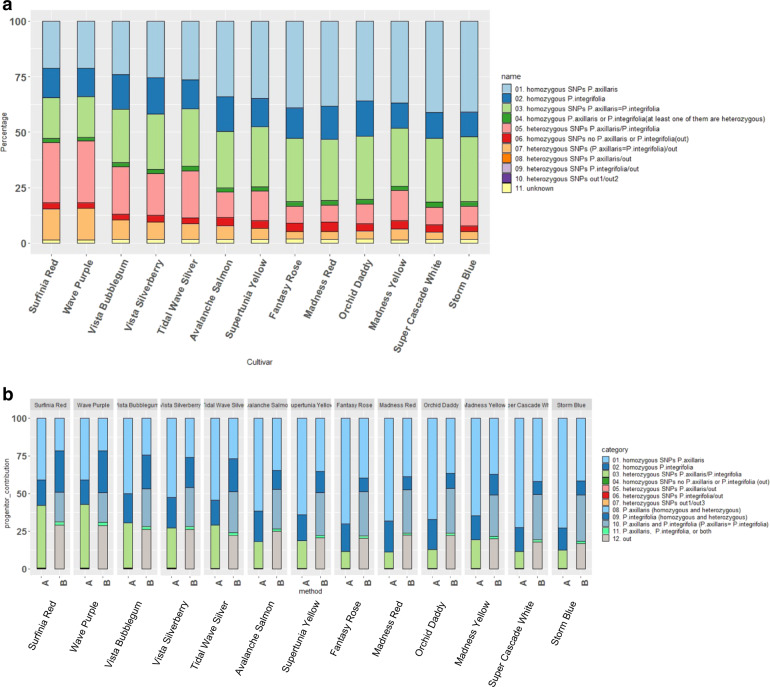
Genetic dissection of the alleles in the petunia cultivars. The categories were derived from the genotypic similarity of the cultivars with *P*. *axillaris*, *P*. *integrifolia*, or other species represented by the percentage of transcriptome-derived SNPs in each genotype category. The estimation was made using the entire set of the SNPs (1,164,566 SNPs). **a** The estimated SNP proportion was calculated following Bombarely et al.^5^. **b** The estimated SNP proportion using the “exact parents” model (**a**) and the “pure parents” model” (**b**). Out: No polymorphisms detected between *P*. *axillaris* and *P. integrifolia*, whereas the cultivar genotypes had a different allele at the locus. Unknown: Allele origin could not be determined due to missing genotyping data in either *P*. *axillaris* or *P*. *integrifolia*. The cultivars were arranged according to the PCA similarity on PC1 (Supplementary Fig. 1).

The genetic background for each cultivar was further parsed (with 1,164,566 SNPs) according to similarity with the progenitor species *P*. *axillaris* and *P. integrifolia* following two methods: (1) the method of Bombarely et al.^5^ (approach 1); and (2) a two-model thresholds approach (approach 2) (Fig. 2). Following Bombarely et al.^5^, genetic dissection showed that the total percentage of homozygous SNPs ranged from 54.5% to 83.8% across the cultivars, whereas the total percentage of homozygous alleles derived from *P*. *axillaris* and *P*. *integrifolia* in these cultivars ranged from 34.0% to 53.3% (Fig. 2a).

Most of the alleles in the cultivars were derived from the progenitor species. The majority of heterozygous SNPs in the cultivars were heterozygous alleles of *P*. *axillaris*/*P*. *integrifolia* (7.4–27.7%), followed by heterozygous SNPs with one nucleotide from the common allele of *P*. *axillaris* and *P*. *integrifolia* and another nucleotide other than the current *P*. *axillaris* or *P*. *integrifolia*, which were named “out” genotypes. The “out” genotypes (3.3–14.3%) could come from another species or from other *P*. *axillaris* and *P*. *integrifolia* genotypes than those used in this study.

A higher percentage of *P*. *axillaris* alleles (34.7–45.2%) were detected in the cultivars than *P. integrifolia* alleles (15.5–26.9%) when the number of *P*. *axillaris* and *P*. *integrifolia* alleles in the heterozygous *P*. *axillaris*/*P*. *integrifolia* SNPs were set as 50% of those in homozygous SNPs (*P*. *axillaris/P*. *axillaris or P*. *integrifolia/P*. *integrifolia*), and the loci where *P*. *axillaris* and *P*. *integrifolia* showed no polymorphisms were excluded (25.1–31.1%). The percentage of *P*. *axillaris* alleles was higher in the upright cultivars compared to the trailing types, while the percentage of *P*. *integrifolia* alleles was higher in the trailing cultivars compared with the upright cultivars.

Interestingly, alleles not derived from either progenitor species (“out” alleles) were detected across all cultivars, which indicates that there could be allele introgression events from other species during cultivar development (Fig. 2 and Supplementary Table 2). Those alleles may comprise 4.5–9.9% of the SNPs in the transcriptome. This estimation was based on the assumption that the heterozygous “out” SNPs [including *P*. *axillaris*/out, *P*. *integrifolia*/out, and (*P*. *axillaris* = *P*. *integrifolia*)/out] had 50% fewer “out” alleles compared to homozygous “out” SNPs (out/out or out1/out2). ‘Wave Purple’ and ‘Surfinia Red’ had the largest percentage of “out” alleles among these cultivars (~9.9%).

For the *P*. *axillaris* genotype used in this study, approximately 59% of the SNPs showed “*P*. *axillaris* only” alleles, and for the *P*. *integrifolia* sample, about 35% SNPs showed “*P*. *integrifolia* only” alleles. In addition, *P*. *axillaris* and *P*. *integrifolia* shared approximately 35% common homozygous alleles.

It has been reported that the first petunia hybrids were produced by multiple crosses from different accessions of the two parent clades^2,16^. The exact genotypes of the wild species from which the current petunia cultivars were derived are unknown. In this study, both *P*. *axillaris* and *P*. *integrifolia* accessions had some heterozygosity (the heterozygosity of *P*. *integrifolia* was high). Bombarely et al.^5^ assumed that both parental accessions were 100% homozygous, which probably will not accurately represent the true genetic components of the cultivars. Therefore, a “two-threshold” method was implemented to estimate the range of the genetic proportion in the cultivars derived from the progenitor species. To further estimate the range of genomic components from the progenitor species, we developed a model assuming two “extreme statuses” of the *P*. *axillaris* and *P*. *integrifolia* genotypes: (1) the hypothetical progenitor species are 100% homozygous, with no common alleles (*P*. *axillaris* is 0/0 and *P*. *integrifolia* is 1/1, and no genomic similarity); and (2) the *P*. *axillaris* and *P*. *integrifolia* samples used in this study (with the current heterozygosity) are the exact progenitor genotypes from which these cultivars were developed (Fig. 2b). Under the first assumption, in the cultivars, the contribution of *P*. *axillaris* alleles ranged from 62.0% to 78.9%, and the contribution from *P*. *integrifolia* ranged from 20.8% to 37.6%. In addition, <1% SNPs had alleles that came from species other than *P*. *axillaris* or *P*. *integrifolia* (“out” alleles). Under the second assumption, 21.6–41.6% of the *P*. *axillaris* SNPs and 8.8–27.8% of *P*. *integrifolia* SNPs were detected in the cultivars. In addition, 16.8–28.9% of the SNPs were “out” alleles and 19.8–30.5% of the SNPs shared common alleles between *P*. *axillaris* and *P*. *integrifolia*. Under both assumptions, *P*. *axillaris* SNP alleles were estimated to comprise a higher percentage for the upright cultivars than the trailing cultivars while the *P*. *integrifolia* and “out” alleles were higher in the trailing than the upright cultivars. Also, under the second assumption, the estimated percentage of *P*. *axillaris* and *P*. *integrifolia* shared alleles was lower in the trailing cultivars than the upright types. The estimated ancestral alleles contribution from approach 1 (using the real dataset) is within the estimated progenitor proportion threshold using the hypothetical ancestor genotype model (approach 2).

### The distribution of the ancestral alleles across the petunia genome

**Fig. 3:**
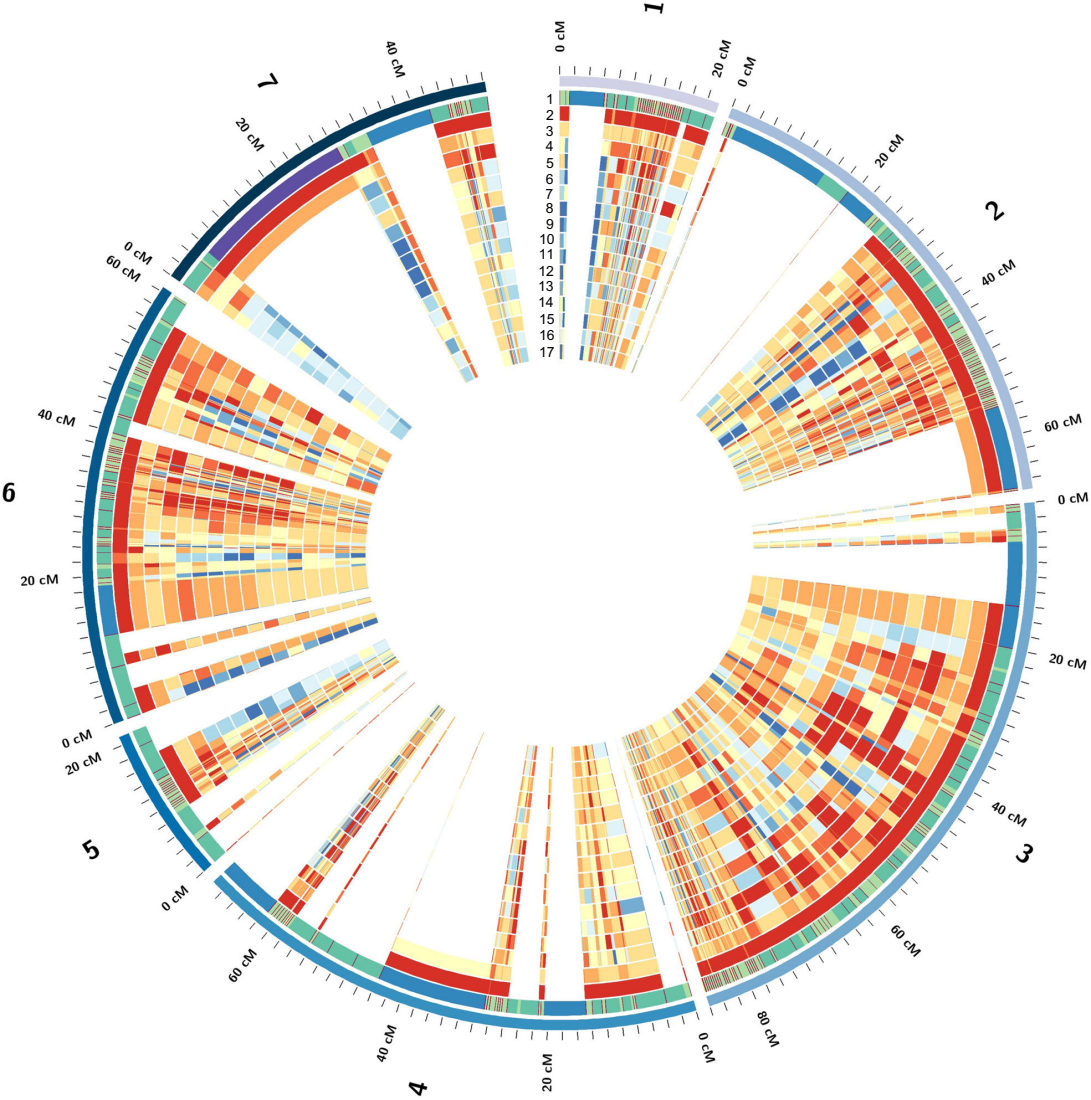
Overall distribution of the genotypic similarity of the entire genotype panel with *P. axillaris* on the *P. axillaris* × *P. exserta* (AE) genetic linkage map. Ring 1 represents the genetic distance between markers on the AE map, while rings 2–17 represent the location and relative abundance of all *P. axillaris* alleles in: (2) *P. axillaris*, (3) *P. integrifolia*, (4) *P*. *exserta*, (5) Madness Yellow, (6) Storm Blue, (7) Super Cascade White, (8) Fantasy Rose, (9) Madness Red, (10) Orchid Daddy, (11) Supertunia Yellow, (12) Avalanche Salmon, (13) Tidal Wave Silver, (14) Wave Purple, (15) Surfinia Red, (16) Vista Bubblegum, and (17) Vista Silverberry. Dark red indicates highest similarity to *P. axillaris* while dark blue indicates lowest similarity to *P. axillaris*. The three “outer layers” are the three wild species. The cultivars were arranged, from outer to inner ring, according to their similarity with *P*. *axillaris*.

To dissect the genome-wide distribution of the ancestor origins for the cultivars, each locus was calculated for the percentage of similarity with the genotype of *P*. *axillaris* following Bombarely et al.^5^. The similarity ratio was then projected onto the high-density genetic linkage map according to the corresponding locations of the transcripts on the *P*. *axillaris* scaffolds and the genotyping-by-sequencing (GBS)-SNP locations on the AE (*P. axillaris* × *P. exserta*) genetic linkage map that we generated previously^6,11^. Overall, 1345 unique transcripts were placed on the AE map corresponding to 1978 GBS-SNP loci, which was 31.4% of the total SNPs on the AE map (Fig. 3). These 1345 transcripts had 65,170 SNP loci, and the percentage of similarity with *P*. *axillaris* was calculated for each transcript of each cultivar. A very small portion of transcripts (32 transcripts) were mapped to multiple locations on the AE map, yet most of these locations could be combined because of their short genetic distances (located within 1 cM). A few transcripts (<10) were mapped to locations >20 cM apart on the same linkage group. In this case, both locations were kept and the percentage of similarity with *P*. *axillaris* were compared with the adjacent mapped regions to gain a comprehensive understanding of the overall progenitor background distribution on the linkage map.

Some consistent patterns could be detected from the genome-wide distribution of genetic similarity with the hypothetical progenitor species *P*. *axillaris* (Fig. 3). *P. axillaris* had small regions of heterozygosity that tended to distribute toward the end of each linkage group. The *P. integrifolia* genotype had high heterozygosity and most regions on the linkage group were heterozygous. *P. exserta* had overall high similarity with *P*. *axillaris*. The regions with less identity were either heterozygous regions or regions with alleles introgressed from “out” (other) species or genotypes. We detected regions across the cultivar panel where both *P*. *axillaris* and *P. integrifolia* samples had high similarity with *P*. *axillaris* (red color), while all cultivars had low similarity (blue color). These could be the regions where the “out” alleles were introgressed.

No chromosome (linkage group) with exclusive genetic similarity with either of the progenitor species was detected for the cultivars. The percentage of similarity with *P*. *axillaris* was lower in the trailing cultivars compared with upright cultivars. Consistent with the principal component analysis (PCA) analysis (Supplementary Fig. 1), the cultivars ‘Vista Bubblegum’ and ‘Vista Silverberry’ showed almost identical ancestral genetic contribution pattern on the linkage maps, indicating a closely related breeding background.

### The wild species introgression pattern in trailing and upright cultivars

**Fig. 4:**
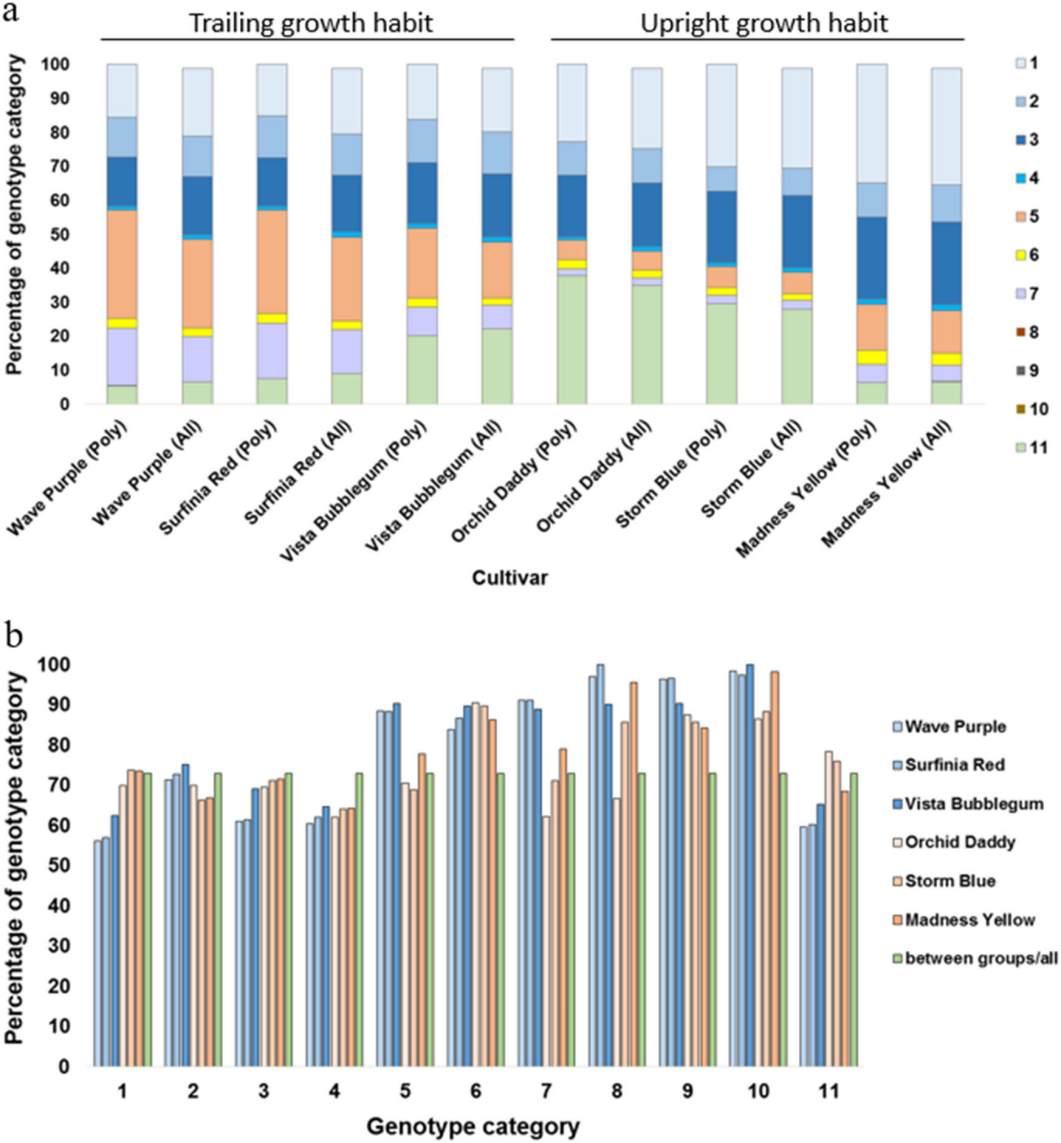
Genotype category 7 [heterozygous (*P. axillaris* = *P. integrifolia*)/out] SNPs were enriched between the trailing type cultivars and the upright cultivars. **a** Overview of the distribution of the percentage of SNPs between the trailing cultivars (‘Wave Purple’, ‘Surfinia Red’, and ‘Vista Bubblegum’) and the upright cultivars (‘Orchid Daddy’, ‘Strom Blue’, and ‘Madness Yellow’) dissected into each genotype category (Bombarely et al.^5^), compared with the percentage of SNPs in the entire dataset. **b** Comparison of the percentage in each genotype category using SNPs between the trailing cultivars (blue), the upright cultivars (orange), and the percentage of SNPs using the entire dataset (green). The genotyping categories were as in Fig. 2a. The cultivars on the *x*-axis were arranged according to their growth habit (trailing: ‘Wave Purple’, ‘Surfinia Red’, and ‘Vista Bubblegum’; upright: ‘Orchid Daddy’, ‘Strom Blue’, and ‘Madness Yellow’).

SNPs between the representative trailing and upright cultivar groups were distributed on 95.8% of the *P*. *axillaris* transcriptome (839,794 SNPs located on 31,613 transcripts). For all the cultivars in the representative growth habit group, the proportion of each genotype category did not seem to change significantly in comparison with their proportions in the entire SNP set (Fig. 4a). Since neither *P. axillaris* nor *P. integrifolia* have a trailing growth habit, we assumed that the trailing growth habit was most likely contributed by the “out” alleles introgressed from another species. The “out” alleles are distributed in genotyping categories 6, 7, 8, 9, and 10. Of these, the vast majority (over 99.9%) were category 6 and 7 SNPs. When comparing the proportion of “enriched SNPs” (SNPs that are only polymorphic between the trailing and upright cultivar groups/total number of SNPs) in each genotyping category for each cultivar, the proportion of category 7 [heterozygous SNPs (*P*. *axillaris* = *P*. *integrifolia*)/out, Supplementary Table 1] was significantly increased only for the trailing cultivars, in comparison with the upright cultivars, and had an overall higher percentage than the expected average proportion (Fig. 4b). The enriched category 7 genotypes in the trailing cultivars could indicate that, in this category (genotyping pattern), alleles introgressed from other petunia species might be related to the horizontal growth habit of trailing petunia cultivars.

**Fig. 5:**
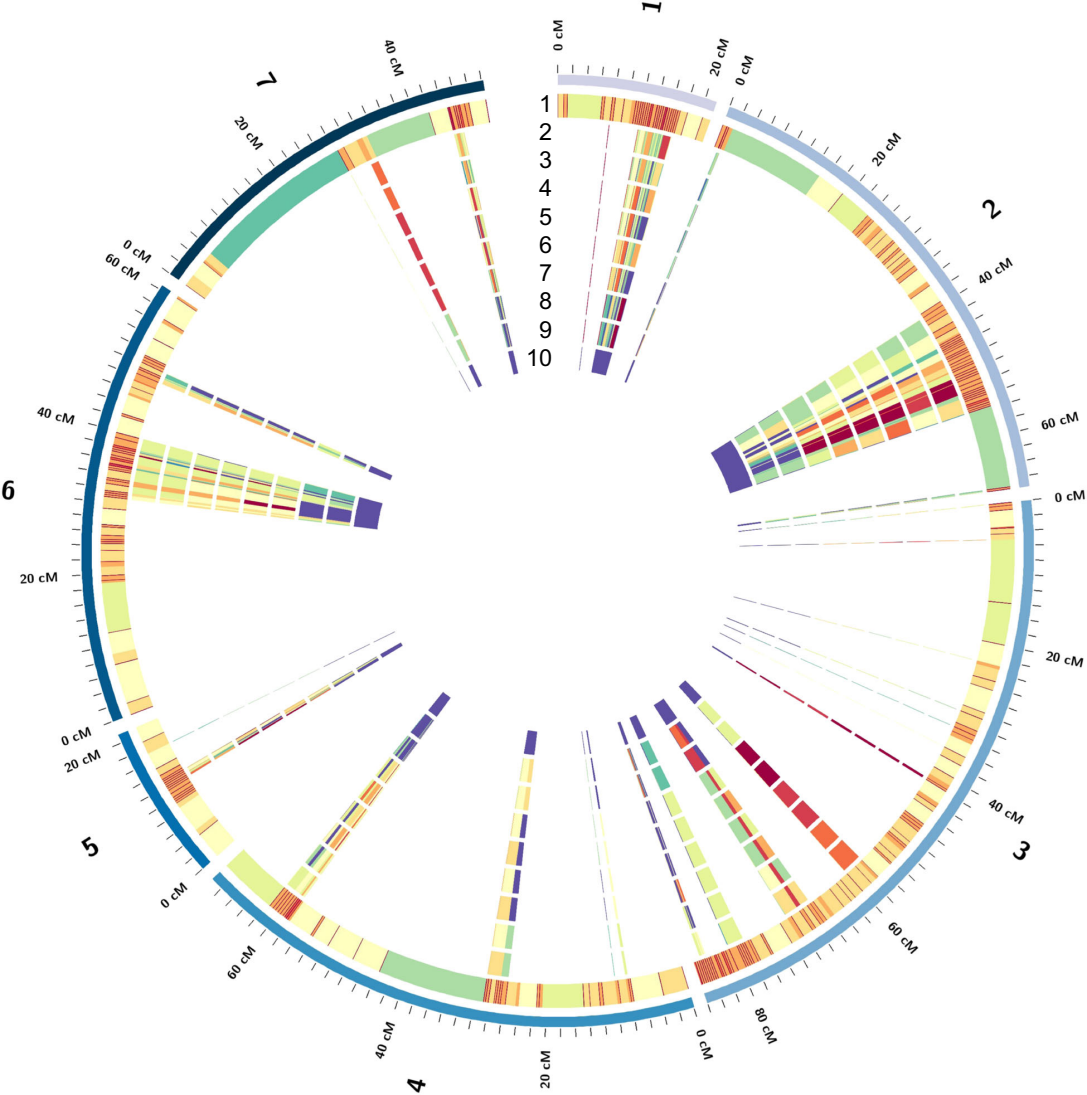
Overview of distribution of polymorphic “out-group” SNPs between trailing-type cultivars and upright cultivars on the *P. axillaris* × *P. exserta* (AE) genetic linkage map. Ring 1 represents the genetic distance between markers on the AE map, while rings 2–10 represent the location and the frequency of introgressed alleles (SNPs) representing (2) all “out” alleles in the trailing group, (3) all category 7 [heterozygous (*P. axillaris* = *P. integrifolia*)/out] alleles in the trailing group, (4) all “out” alleles in Wave Purple, (5) all category 7 alleles in Wave Purple, (6) all “out” alleles in Surfinia Red, (7) all category 7 alleles in Surfinia Red, (8) all “out” alleles in Vista Bubblegum, (9) all category 7 alleles in Vista Bubblegum, and (10) all “out” alleles in the upright cultivar group B. The cultivars were arranged according to growth habit.

About 19.2% of the *P*. *axillaris* transcriptome (6323 transcripts) had SNPs that were polymorphic between the trailing and upright cultivars with alleles introgressed from a third species (“out” alleles from genotype category 6, 7, 8, 9, or 10). Of those, 2463 transcripts (7.5% of the *P*. *axillaris* transcriptome assembly) had category 7 SNPs in every representative trailing cultivar, while no category 7 SNPs were present in any upright cultivar. To characterize the genome-wide “third” species introgression patterns, which might elucidate regions correlated with the petunia trailing growth habit, we first placed transcripts that had any of the “out” genotype categories (genotype categories 6, 7, 8, 9, and 10) and were polymorphic between the trailing and upright cultivar groups on the AE linkage map (Fig. 5). Overall, 323 transcripts were placed on the AE linkage map. We then examined the regions where genotype 7 alleles were present only in the trailing cultivars, which could harbor genes related to the trailing growth habit. The Circos plot showed that the outgroup introgression regions were present on every chromosome. The location and abundance of introgressed “out” alleles on the AE map indicated that the introgressions tended to concentrate in high recombination frequency regions of the genome, and the abundance of overall “out” alleles and the 7 type-only alleles were consistent. The introgression frequency varied slightly among the three trailing type cultivars, with ‘Wave Purple’ and ‘Surfinia Red’ having overall both higher “out” and category 7 allele frequencies than ‘Vista Bubblegum’ (Fig. 5).

Genes involved in petunia axillary meristem outgrowth from previous reports were then placed within or adjacent to these genotype 7-only regions (Supplementary Table 3). Of these, we found that *DAD1*/*CCD8* (AY746977.1) was located on chromosome 7 on scaffold Peaxi162Scf00227, which was near several transcripts harboring genotype 7-only alleles with the trailing cultivars. In addition, a *P*. ×*hybrida* TCP transcription factor 3 (*TCP3*) (KR002105.1) was found located on chromosome 3 and on the same scaffold that has transcripts within the outgroup allele introgression region, but both “out” alleles and the genotype 7 alleles were only present in ‘Wave Purple’ and ‘Surfinia Red’. For the genes with corresponding transcripts, the outgroup genotypes and the genotype 7 category distribution was further investigated. However, no transcripts had genotype 7 unique SNPs only present in the trailing cultivars (some of these were due to missing data in some of the cultivars).

For transcripts that had category 7 SNPs in every representative trailing cultivar, while no category 7 SNPs were present in any upright cultivars, gene ontology (GO) enrichment showed that the GO terms were enriched in the regulation of biological process and cellular process, many related to developmental process and response to stimulus (heat, chemical, light, etc.). For the molecular functions, we found a considerable number of transcription regulation and catalytic activity (Supplementary Table 4). These GO terms were similar with previously reported GO terms associated with growth habit in olive (*Olea europaea* L.)^17^. Longer internodes usually associated with trailing type while upright types had shorter internodes. In this set of genes, the carbohydrate metabolic processes and their regulation were enriched, consistent with the report on internode length in *Lagerstroemia*^18^. A total of 709 of these transcripts (unigenes) were annotated with the KO (Kyoto Encyclopedia of Genes and Genome (KEGG) Ortholog) database, and the most representative pathways were “Metabolic pathways” (98), “Biosynthesis of secondary metabolites” (44), and “RNA transport” (21) (Supplementary Table 5).

## Discussion

The origin of *P*. ×*hybrida* was proposed to be from the breeding of only two species, one with white flowers (*P*. *axillaris*) and the other with purple flowers (*P. integrifolia*)^16^. Early petunia breeding was mainly through selection within the available hybrids^3^. However, by 1970 all cultivars produced a relatively uniform phenotype (large flowers on compact plants). Therefore, during the 1990s, petunia breeding programs started to introduce alleles from other *Petunia* species or old cultivars^3^. This process introduced a new phenotype—prostrate or trailing growth habit. The short yet complex breeding history shaped the current genome of petunia cultivars, which exhibit wide morphological variation^19^. Until now, the genetic background of commercial petunia cultivars remained uncharacterized. Elucidating the genetic diversity of petunia cultivars and inferring their genomic background from the progenitor species would facilitate the understanding of petunia breeding history and identification of the causal variants/genomic regions associated with important traits, such as stress tolerance and growth habit. In this study, transcriptome-derived SNPs were used to characterize the genetic variation among petunia cultivars.

*P. integrifolia* and *P*. *axillaris* were the hypothetical ancestors of petunia cultivars. *P. exserta* is not considered as a progenitor species^2^, but it may be useful for introgression of genes for novel traits such as flower color and shape, and growth habit, into the cultivated petunia^3,20^. *P. integrifolia* has five taxa and three of them could be a progenitor of the garden petunia^3,4,21,22^. *P. exserta* is the only ornithophilous species of the genus, with red flowers, protruded anthers, and stigma. Similar to previous findings that *P*. *exserta* and *P*. *axillaris* are closely related species^22–24^, in this study, >70% of the transcriptome-derived SNPs between the two species were monomorphic. The close phylogenetic relationship and the similar genetic distribution in PCA plots (Supplementary Fig. 1a) both support that the divergence between the two species is recent. On the genetic linkage map, we found regions harboring different genetic background between *P*. *exserta* and *P*. *axillaris*. These regions were detected on almost all linkage groups (chromosomes) and could contain important genes for habitat shift and floral traits or other factors involved in genetic isolation of these species. A number of recent reports and model predictions indicate the accumulation of differentiations/barrier genes among species in regions of low/restricted recombination^25,26^, including for *Petunia*^27^.

For the petunia cultivars, a range of genetic diversity was detected. On the PCA plot (Supplementary Fig. 1a), the three wild species were distributed toward the ends of both axes, while the cultivars tend to cluster in the middle. This could suggest that the majority of petunia cultivars behaved as true hybrids of the hypothetical progenitor species and had large genetic contribution from each of the two species.

Unlike many major crops, no signature of breeding was detected among petunia cultivars, possibly because of the short breeding history and the diversified breeding objectives. On the other hand, this could also be due to the decreased statistical power from the small sample size, even when potential candidate SNPs were identified by searching for SNPs with absolute alpha values (which indicate the strength and direction of selection) ranked within the top 5 and 10%.

We found that the *P*. ×*hybrida* genome contains genetic contribution from *P*. *axillaris*, *P. integrifolia*, and one or more unknown ancestors. This finding is consistent with previous research that showed both progenitor species and possibly other species contributed to the current genome of *P*. ×*hybrida*^5^. However, our study also showed that the proportion of contribution for each progenitor species varied across cultivars, though all cultivars contained a higher proportion of *P. axillaris*-unique alleles compared to *P. integrifolia*-unique alleles (Fig. 2). In addition, cultivars with similar proportions of *P*. *axillaris* and *P. integrifolia* contribution also had higher heterozygosity than those with a higher proportion of *P. axillaris* alleles. Heterozygosity levels for the traditional upright varieties averaged 10–15% (Fig. 1), which was surprisingly low considering that most are F_1_ hybrids. This suggests that the commercial *P*. ×*hybrida* germplasm pool may be of quite limited diversity. Identifying the genetic regions contributed by each of the progenitor species may provide a landscape of the recombination pattern across the petunia cultivar panel. However, at present, the genetic location on the linkage map does not directly correspond to the physical distance, as the recombination rate (cM/Mb) may vary on the genome and for each genotype.

In previous research on the *P*. ×*hybrida* genome, the majority of the genetic contribution was from *P*. *axillaris*^5^. A possible explanation for this difference could be the different representative *P*. ×*hybrida* genotypes used in the study. Commercial cultivars were used here, whereas three laboratory accessions were used formerly^5^. For the three laboratory lines, the cultivar ‘Mitchell’ was developed from a doubled haploid generated from a hybrid between *P*. *axillaris* and the backcross of the cultivar “Rose of Heaven” and *P*. *axillaris* F_1_ hybrid. Therefore, a high percentage of the *P*. *axillaris* genes was expected^28^. In the other two accessions, both had >10 generations of inbreeding, which could contribute to the genetic background bias toward one parent. We did not have enough resolution to further investigate the SNP distribution pattern at a gene level on the cultivar panel. Therefore, we could not verify on the petunia cultivars the previous findings that conserved blocks of SNPs similar to *P*. *axillaris* and other blocks similar to *P. integrifolia* between the previously reported genotypes could be due to gene conversion or random repair of heteroduplexes^5^.

The trailing cultivars have a short breeding history, dating back only to the 1990s^3^. The initial trailing petunias were reportedly developed by crossing *P*. ×*hybrida* with an unknown *Petunia* species^29^. Therefore, the prostrate or trailing phenotype was likely introduced through alleles of another wild species. In this study, SNPs of genotype category 7 [(*P*. *axillaris* = *P*. *integrifolia*)/out] were enriched in the representative trailing cultivars and thus added a piece of evidence that the trailing phenotype might be introduced from a third species. Considering all 14 petunia species are freely crossable with *P*. ×*hybrida*^30^, and *P*. *altiplana* has been reported to be “widely spreading and often forming a mat-like structure”^31^, unique within the genus^2^, we suspect that the trailing alleles in the cultivars were introgressed from *P*. *altiplana*.

However, if the trailing cultivars were the only type that had introgressed alleles from other species, the “out” alleles should be present only in these cultivars, which is inconsistent with the current findings. This could be due to: (1) the fact that exact progenitor genotypes for the petunia cultivars are unknown, therefore, some of the “true progenitors” alleles were not present in the current *P*. *axillaris* and *P*. *integrifolia* genotypes, which may have biased the genotype category estimation. It is also possible that the proportion of “out” alleles were overestimated; (2) the complex breeding history of petunia cultivars might have introduced alleles from other species than the two main progenitor species. Nevertheless, the most likely alleles for the trailing growth habit should be the “out” alleles that were only present in the trailing cultivars. Genome-wide comparisons of representative trailing cultivars with the compact cultivars showed that the introgressed “out” alleles were not restricted to a few discrete regions, instead, they were widespread and mostly heterogeneous loci across the genome.

## Materials and methods

### Transcriptome sequencing and read collection for petunia cultivars

A panel of 13 petunia cultivars with diverse growth habits was selected for transcriptome sequencing. Three of the cultivars—‘Wave Purple’ (trailing type), ‘Surfinia Red’ (trailing type), and ‘Madness Yellow’ (upright type), were selected to generate a reference transcriptome for *P*. ×*hybrida*. For these cultivars, tissues were collected from combined stages of floral development (from ca. 5 mm long buds through early fruit development) and leaves at different developmental stages (from ca. 1/10th up to 1/3rd of final expanded leaf length) pooled from five 10-week-old plants per cultivar and immediately frozen in liquid N_2_. For each of these cultivars, RNA extraction was carried out on pooled flower and leaf samples separately. To investigate the genomic background and genetic diversity of petunia cultivars, an additional ten cultivars were sequenced. For these ten cultivars, only leaf tissues were collected (same stages as above) and pooled for RNA extraction, again from five plants per cultivar. Of these cultivars, ‘Supertunia Yellow’, ‘Supertunia Vista Bubblegum (Vista Bubblegum)’, ‘Supertunia Vista Silverberry (Vista Silverberry)’, and ‘Tidal Wave Silver’ have a mounding/trailing habit; ‘Avalanche Salmon’ has a mounded habit; and ‘Fantasy Rose’, ‘Madness Red’, ‘Orchid Daddy’, ‘Storm Blue’, and ‘Super Cascade White’ have an upright habit (Table 1).

RNA was extracted using the Trizol® reagent (Life Technologies) following the manufacturer’s instructions. Forty micrograms of RNA was treated for DNA contamination with RNase-free DNase set (Qiagen). DNase-treated RNA was purified with the RNeasy® MinElute Cleanup Kit (Qiagen). RNA yield and quality were determined by Agilent 2100 BioAnalyzer RNA 6000 Pico chip (Agilent Technologies) with threshold RNA integrity number values ≥8.0. A TruSeq RNA Sample Preparation kit (Illumina) was used to construct the cDNA libraries. The six libraries of the leaf samples and flower samples for ‘Wave Purple’, ‘Surfinia Red’, and ‘Madness Yellow’ were pooled in one lane and sequenced on the Illumina HiSeq 2500 sequencer with 100 nt paired-end at the Michigan State University Research Technology Support Facility (RTSF; http://rtsf.msu.edu/; East Lansing, MI). For the other ten cultivars, the samples were barcoded and run on one lane of SE50 Illumina High Output flow cell at the Michigan State University RTSF. Sequences were filtered and trimmed with Trimmomatic with the corresponding functions for paired- and single-end reads, respectively^32^. Both raw and cleaned reads were assessed with FastQC v0.11.3 (http://www.bioinformatics.babraham.ac.uk/projects/fastqc/).

### SNP detection, validation, and re-genotyping

Sequencing reads for the cultivar samples and wild species (*P*. *axillaris*, *P*. *exserta*, and *P*. *integrifolia*) reported previously^6^ were mapped to the *P. axillaris* transcriptome assembly using the Burrows–Wheeler Aligner program with default values for both single- and paired-end reads^6,33^. Duplicate reads were removed after the initial alignment to eliminate reads that mapped to the same position of the transcriptome. Duplicate removal was performed for the aligned reads using picardTools/1.89 (broadinstitute.github.io/picard). The subsequent local realignment to correct misalignments due to the presence of indels was performed using the Genome Analysis Toolkit (GATK)^34^. Initial variant calling was performed by HaplotypeCaller from GATK with a Phred-scaled confidence threshold of 30. After the initial variant calling, SNP filtering was performed using previously described criteria except with a modification to retain clustered SNPs (SNP clustering filter was removed) and with a minimum total depth of coverage of five for each genotype^6^. Afterwards, SNPs that showed no polymorphisms within the panel were excluded using the filters as previously described^6^.

SNP validation was carried out on 22 randomly chosen SNPs with no missing data on the genotype calling to ensure the maximum genotyping validation efficiency. The primers were designed around SNP loci where at least two species were polymorphic and on unigenes with exons >600 bp. Primer design, PCR conditions, and sequence visualization were as previously described^6^. The initial SNP validation identified that a small portion of the heterozygous genotypes were called as homozygous, therefore for the subsequent analyses, a series of custom genotyping filters were implemented to exclude low-quality reads or ambiguous genotypes and to re-define the genotype using allele read percentage. The filters were written by Python (Python 2.7.9)^35^ with the following criteria: (1) AA: SNPs were called homozygous and assigned to have the same genotype as the reference genotype (*P*. *axillaris*) when the reference reads were ≥80% of the total reads; (2) BB: SNPs were called homozygous alternative alleles if the alternative reads were >80% of the total reads; (3) AB: SNPs were called heterozygous if both the ratios of reference SNP reads to total reads at the loci and the ratio of the first alternative allele reads to entire reads at the loci were >0.3, and the sum of both ratios is ≥0.8. After the genotyping correction, SNPs were filtered to only keep the polymorphic loci.

### Detecting signature of selection in petunia cultivars

To detect the presence of a breeding signature among the cultivars, the level of differentiation at a locus was compared with the level of differentiation across the transcriptome with 74,218 SNPs that had no missing data across the 16 genotypes. The *F*_ST_ outliers (loci deviating from neutral expectations) were detected by BayeScan v2.1 with default parameters^15,36,37^. BAYESCAN v. 2.163 implemented the Bayesian model decomposes *F*_ST_ values into locus-specific components (*α*) and population-specific components (β51). The method is based on a reversible jump MCMC algorithm and calculates the posterior probability of each under selection locus by assuming two alternative models, selection-based model and neutral model. A locus that is significantly more divergent than average is likely been affected by positive or direct selection. BayeScan can perform analyses on very small sample size without introducing bias^15,36,37^. The alpha values generated by BayeScan indicate the strength and direction of selection, with a positive value suggests diversifying selection and negative value suggests balancing or purifying selection^38^.

### Dissecting the genetic background of petunia cultivars

Heterozygosity rate was calculated as the proportion of heterozygous SNPs for each individual (cultivars and the three wild species). Both the entire SNP dataset (1,164,566) and the set of SNPs with no missing data (74,218) were used for the heterozygosity rate estimation.

With the entire dataset (1,164,566 SNPs), two genotype classification approaches were used to understand the genetic background of petunia cultivars and their relationship with the ancestor species. The first approach (approach 1) followed the previous publication of Bombarely et al.^5^ with a slight modification. In brief, the cultivar genotypes were classified into 12 categories (Supplementary Table 1) by their nucleotide similarity with *P*. *axillaris* and *P*. *integrifolia*, where both *P*. *axillaris* and *P*. *integrifolia* were assumed to be homozygous and had different genotypes (0/0 for *P*. *axillaris* and 1/1 for *P*. *integrifolia*). Except that, at loci where *P*. *axillaris* and *P*. *integrifolia* had the same genotype calling, the cultivars with the same genotype as *P*. *axillaris* and *P*. *integrifolia* would be called homozygous *P*. *axillaris* = *P*. *integrifolia*, and any other alleles were categorized as from species other than *P*. *axillaris* and *P*. *integrifolia* (“out” alleles, when no polymorphisms were detected between *P. axillaris* and *P. integrifolia*, whereas the cultivar genotypes had a different allele at the locus). Unknown: Allele origin could not be determined due to the missing genotyping data in either *P. axillaris* or *P. integrifolia*. (Supplementary Table 1). SNPs with unknown genetic background due to missing data were assigned to the “unknown” category. The second approach (approach 2) did not directly estimate the cultivar genetic components, instead, a “thresholds model” was employed to assess the cultivar genotype proportion range for each category. One boundary assumed that the cultivar panels were derived from ancestral species that had exactly the same *P*. *axillaris* and *P*. *integrifolia* genotype as used in this study (where the *P*. *integrifolia* has >50% heterozygosity), therefore any cultivar genotypes that were the same as *P*. *axillaris* or *P*. *integrifolia* would be counted as derived from *P*. *axillaris* or *P*. *integrifolia*, and alleles not found in *P*. *axillaris* or *P*. *integrifolia* were assumed derived from other species (“out”). The second boundary assumed that both *P*. *axillaris* and *P*. *integrifolia* are homozygous, *P*. *axillaris* had the “0/0” genotype, and *P*. *integrifolia* had the “1/1” genotype. In such circumstances, the cultivars were dissected according to their similarity with “0/0” and “1/1”, and consequently the cultivars category classification was independent of the *P*. *axillaris* and *P*. *integrifolia* genotype calling.

Once the genetic background was identified for each cultivar, the percentage of homozygous *P*. *axillaris* SNPs for each transcript was projected to the genetic linkage map generated from an RIL population developed from the crossing of *P*. *axillaris* with *P*. *exserta* (AE map)^11^. The percentage of homozygous *P*. *axillaris* SNPs for a transcript was calculated by first assigning all SNPs for that cultivar on the transcript to a similarity rate based on their genotype categories (Supplementary Table 1). Subsequently, the average similarity was calculated for all SNPs on that transcript. Furthermore, a custom Python script was used to place the transcripts onto the AE map if a SNP on the map was located within the transcript covered range on the genome. Finally, the global percentages of SNP similarity with *P*. *axillaris* were calculated within each recombination interval. On the AE map, regions with low genetic recombinant frequency had only a few GBS-SNPs spanning long genetic distances. To avoid biased estimation, *P. axillaris* genetic similarity was not plotted on these regions. The percentage of SNP similarity with *P*. *axillaris* on the AE linkage groups was visualized using Circos^39^.

### Exploring the genetic components difference for growth habit types in petunia cultivars

Two groups representing different growth habit types were used to study the genetic difference between the trailing and upright types. The trailing type group (Group A) had three cultivars: ‘Wave Purple’, ‘Surfinia Red’, and ‘Vista Bubblegum’; and the upright cultivar group (Group B) included ‘Orchid Daddy’, ‘Storm Blue’, and ‘Madness Yellow’. To inspect what genotype categories have been “enriched” in the trailing-type cultivars, the SNPs that were polymorphic between two groups were dissected into the 12 categories (approach 1), and the proportion of each category in the polymorphic SNP set and the original set were compared for each cultivar. In addition, the percentage of each category in the current polymorphic SNP set compared to the original SNP set were plotted for the cultivars, along with the average proportion of each category of polymorphic SNP in the entire SNP set plotted for comparisons.

After the candidate transcripts were identified (with alleles potentially introgressed from other species that might be related with growth habit), we performed a GO enrichment analysis using topGO package^40^ in R version 3.6 to identify if genes with particular molecular function and biological processes were overrepresented in these transcripts. The GO enrichment employed Fisher’s exact test for statistical significance. The enriched GO terms at *p* value of ≤0.05 were retained. Treemaps were produced to visualize enriched GO categories. Metabolic and the functional biological pathways were identified by mapping transcripts under the significantly enriched Go terms to the KEGG^41^ (http://www.genome.jp/kegg/) by KEGG Automatic Annotation Server^42^ (http://www.genome.jp/kegg/kaas/) with the available manually annotated plant databases and single-directional best hit.

## Supplementary information

Suuplemental information file

Supplemental Table 4

## Data Availability

The raw data for RNA sequencing have been deposited in NCBI Sequence Read Archive (SRA) (SRA accession: PRJNA546569).
