# Supplementary material for: Dissecting genetic diversity and genomic background of Petunia cultivars with contrasting growth habits
Source: Hortic Res. 2020 Oct 1;7:155. doi: 10.1038/s41438-020-00373-2 (PMC7528118; doi:10.1038/s41438-020-00373-2)
Supplement: Supplementary file 1 — Suuplemental information file [file 41438_2020_373_MOESM1_ESM.docx]

**Supplemental Material**

**Supplemental Materials and Methods**

**Assessing the genetic relatedness among the petunia cultivars by phylogenetic and principle component analysis (PCA)**

Initial PCA (principal component analysis) analysis was carried out with PLINK v1.9 ^1^. SNPs with only one alternative allele, and with Phred quality score >20 at individual level were included in principle coordinate analysis (PCA). Afterwards, the pruned set of SNPs was used for the final PCA to avoid the strong influence of SNP clusters ^2^. SNP pruning was carried out by excluding SNPs in strong linkage disequilibrium (based on pairwise genotypic correlation) by PLINK v 1.9 using the command “--indep-pairwise”. To further estimate the contribution from each component, the PCA analysis was then performed by the “smartpca” module of EIGENSOFT package using the EIGENSTRAT stratification correlation method ^3,4^. The significant principle component was tested by Tracy-Widom test from the EIGENSOFT package ^5-7^.

Neighbor joining trees were generated using the pruned set of SNPs for thirteen cultivars and three wild species. The heterozygous genotypes were randomly set to homozygous at the probability of 0.5, and the neighbor-joining tree was generated by TASSEL with default parameters ^8^.

**Supplemental Results**

**Transcriptome genetic diversity of petunia cultivars and the wild species**

To examine the genetic relationship within the cultivars and between cultivars and species, a neighbor-joining phylogenetic analysis and principle component analysis (PCA) were carried out. For the PCA analysis, the first two eigenvectors explained 3.3% and 2.2% of the genetic variation, respectively. The Tracy-Widom test revealed that the contribution from PC1 (3.22%) was significant. The hypothetical progenitor species *P*. *axillaris* and *P*. *integrifolia* located separated from the thirteen cultivars on the first and the second eigenvectors, respectively. On the first eigenvector, most of the cultivars were located between *P*. *axillaris* and *P*. *integrifolia*, except four cultivars: ‘Vista Bubblegum’, ‘Vista Silverberry’, ‘Surfinia Red’ and ‘Wave Purple’ (Suplemental Fig. 1A). On the PCA plot, cultivars with similar growth habits tended to locate closely. For example, the trailing type cultivars ‘Wave Purple’, ‘Surfinia Red’ and ‘Vista Bubblegum’ were located closely. The upright cultivars ‘Madness Yellow’, ‘Storm Blue’ and ‘Super Cascade White’ were located closely. Moreover, ‘Vista Bubblegum’ and ‘Vista Silverberry’ almost overlapped on the PCA plot (Supplemental Fig. 1A), indicating a possibly very similar genetic background, which would not be surprising as they come from the same breeding program.

The Neighbor-Joining (NJ) tree showed some similar genetic relatedness of the genotypes as the PCA plot (Suplemental Fig. 1B). *Petunia* *axillaris* and *P*. *exserta* were in the same clade. *Petunia* *exserta* is the only red flowered species in the genus, but it was not in the same clade with the two red-flowered cultivars ‘Madness Red’ and ‘Surfinia Red’, which may verified the previous hypothesis that *P*. *exserta* was not the parent of red commercial hybrids ^10,11^. These results should be interpreted with some caution as the heterozygous SNPs might bias the phylogenetic estimation ^12,13^.

**Supplemental Tables**

Supplementary Table 1. Cultivar genotyping categories.

| Category No. | Cultivar genotyping |
| --- | --- |
| 1 | homozygous SNPs *P*. *axillaris* |
| 2 | homozygous *P*. *integrifolia* |
| 3 | homozygous SNPs *P*. *axillaris* = *P*. *integrifolia* |
| 4 | homozygous *P*. *axillaris* or *P*. *integrifolia* (at least one hetero) |
| 5 | heterozygous SNPs *P*. *axillaris* / *P*. *integrifolia* |
| 6 | homozygous SNPs no *P*. *axillaris* or *P*. *integrifolia* (out) |
| 7 | heterozygous SNPs (*P*. *axillaris* = *P*. *integrifolia*)/out |
| 8 | heterozygous SNPs *P*. *axillaris* /out |
| 9 | Heterozygous SNPs *P*. *integrifolia* /out |
| 10 | Heterozygous SNPs out1/out2 |
| 11 | Missing |
| 12 | Unknown |

Supplementary Table 2. Distribution of “out” SNPs in the cultivars

| Cultivar | Total No. of Out SNPs | Total No. of SNPs | %of total SNPs | transcripts | No. of scaffolds | Total transcripts | % of total transcripts | Ave No. of out SNPs/transcript |
| --- | --- | --- | --- | --- | --- | --- | --- | --- |
| Wave Purple | 185435 | 1088101 | 17.04 | 25128 | 1405 | 32994 | 76.16 | 7.38 |
| Surfinia Red | 178785 | 1058931 | 16.88 | 24731 | 1400 | 32994 | 74.96 | 7.23 |
| Avalanche Salmon | 92735 | 921557 | 10.06 | 19045 | 1326 | 32994 | 57.72 | 4.87 |
| Tidal Wave Silver | 80390 | 827770 | 9.71 | 18567 | 1340 | 32994 | 56.27 | 4.33 |
| Fantasy Rose | 51067 | 728436 | 7.01 | 13987 | 1263 | 32994 | 42.39 | 3.65 |
| Madness Red | 66806 | 843758 | 7.92 | 15726 | 1268 | 32994 | 47.66 | 4.25 |
| Orchid Daddy | 53121 | 758773 | 7.00 | 14110 | 1240 | 32994 | 42.77 | 3.76 |
| Storm Blue | 51914 | 838344 | 6.19 | 13858 | 1227 | 32994 | 42.00 | 3.75 |
| White Supertunia | 56303 | 813151 | 6.92 | 14215 | 1209 | 32994 | 43.08 | 3.96 |
| Supertunia Yellow | 72667 | 860933 | 8.44 | 17223 | 1313 | 32994 | 52.20 | 4.22 |
| Bubblegum | 104454 | 905520 | 11.54 | 20923 | 1357 | 32994 | 63.41 | 4.99 |
| Vista silverberry | 87687 | 814339 | 10.77 | 19243 | 1336 | 32994 | 58.32 | 4.56 |
| Madness Yellow | 96231 | 1086932 | 8.85 | 17946 | 1272 | 32994 | 54.39 | 5.36 |

Supplementary Table 3. Location of genes involved in axillary meristem outgrowth.

| Petunia gene names | Petunia genes accession No. | Corresponding *P*. *axillaris* transcripts | Chromosome | Genetic location (cM) | Genome scaffold | Start | End |
| --- | --- | --- | --- | --- | --- | --- | --- |
| TCP2 | KR002104.1 Petunia x hybrida TCP transcription factor 2 (TCP2) mRNA | Locus_37490_Transcript_1/1_Confidence_1.000_Length_1518 | 1 | 11.637 | Peaxi162Scf00367 | 862637 | 863788 |
| SHO | AF346892.1 Petunia x hybrida Sho gene | Locus_22755_Transcript_1/1_Confidence_1.000_Length_1004 | 1 | 11.935 | Peaxi162Scf00753 | 622285 | 623337 |
| PhAT1 | JQ654485.1 Petunia x hybrida transposon PhAT1 | Locus_15063_Transcript_1/1_Confidence_1.000_Length_2275 | 2 | 55.591 | Peaxi162Scf00035 | 2088609 | 2093139 |
| TCP1 | KR002103.1 Petunia x hybrida TCP transcription factor 1 (TCP1) | Locus_34741_Transcript_1/1_Confidence_1.000_Length_1582 | 3 | 84.638 | Peaxi162Scf00086 | 565892 | 567112 |
| TCP3 | KR002105.1 Petunia x hybrida TCP transcription factor 3 (TCP3) | Locus_2090_Transcript_2/3_Confidence_0.667_Length_2027 | 3 | 25.162 | Peaxi162Scf00015 | 735479 | 737065 |
|  | AB035093.1 Petunia x hybrida mRNA for lateral shoot inducing factor | N/A | 3 | 84.345 | Peaxi162Scf00481 | 397280 | 398212 |
| DAD2 | JQ654486.1 Petunia x hybrida DAD2 (DAD2) gene | Locus_14260_Transcript_1/2_Confidence_0.750_Length_1640 | 4 | 28.768 | Peaxi162Scf00081 | 1829372 | 1832716 |
| DAD3 | mRNA FJ790878 Petunia x hybrida carotenoid cleavage dioxygenase 7 (CCD7) gene(DAD3) | N/A | 6 | 35.192 | Peaxi162Scf00377 | 794991 | 799463 |
| DAD1 | AY746977.1 Petunia x hybrida Dad1/CCD8 mRNA | N/A | 7 | 45.055 | Peaxi162Scf00227 | 716530 | 720427 |

SHO, Zubko et al. (2002)^14^; DAD1, Napoli and Ruehle (1996)^15^, Snowden et al. (2005) ^16^; DAD2, Napoli and Ruehle (1996); DAD3, Napoli and Ruehle (1996);TCP1, TCP2,TCP3, Drummond et al.(2015)^17^

Supplementary Table 5. KO annotation classification frequencies

| Pathway hierarchy1 | Pathway Hierarchy2 | Gene(transcript) number |
| --- | --- | --- |
| Metabolism | Carbohydrate metabolism | 70 |
| Metabolism | Energy metabolism | 5 |
| Metabolism | Lipid metabolism | 32 |
| Metabolism | Nucleotide metabolism | 10 |
| Metabolism | Amino acid metabolism | 30 |
| Metabolism | Metabolism of other amino acids | 14 |
| Metabolism | Glycan biosynthesis and metabolism | 17 |
| Metabolism | Metabolism of cofactors and vitamins | 12 |
| Metabolism | Metabolism of terpenoids and polyketides | 13 |
| Metabolism | Biosynthesis of other secondary metabolites | 29 |
| Metabolism | Xenobiotics biodegradation and metabolism | 14 |
| Genetic Information Processing | Transcription | 25 |
| Genetic Information Processing | Translation | 56 |
| Genetic Information Processing | Folding, sorting and degradation | 51 |
| Genetic Information Processing | Replication and repair | 30 |
| Environmental Information Processing | Membrane transport | 4 |
| Environmental Information Processing | Signal transduction | 162 |
| Cellular Processes | Transport and catabolism | 68 |
| Cellular Processes | Cell growth and death | 31 |
| Cellular Processes | Cellular community - eukaryotes | 14 |
| Cellular Processes | Cellular community - prokaryotes | 5 |
| Cellular Processes | Cell motility | 9 |

**Supplemental Figure**


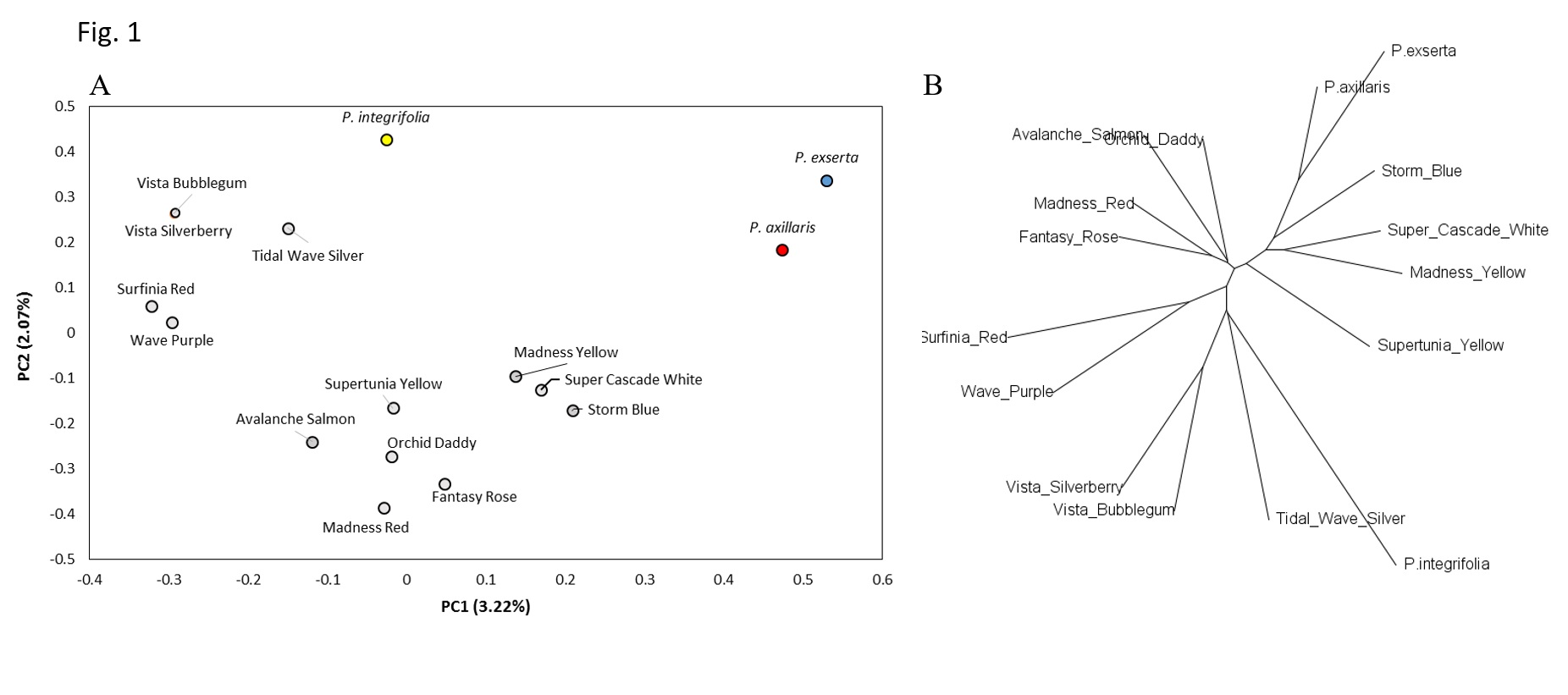


Supplementary Figure 1. Principle component analysis (PCA) using pruned transcriptome derived SNPs for the 13 cultivars and three wild species (A); Neighbor-joining tree with pruned SNPs for the 13 cultivars and three wild species (B).

**References**

1 Purcell, S. *et al.* PLINK: a tool set for whole-genome association and population-based linkage analyses. *Am J Hum Genet* **81**, 559-575, doi:10.1086/519795 (2007).

2 Laurie, C. C. *et al.* Quality control and quality assurance in genotypic data for genome-wide association studies. *Genet Epidemiol* **34**, 591-602, doi:10.1002/gepi.20516 (2010).

3 Patterson, N., Price, A. L. & Reich, D. Population structure and eigenanalysis. *PLoS Genet* **2**, e190, doi:10.1371/journal.pgen.0020190 (2006).

4 Price, A. L. *et al.* Principal components analysis corrects for stratification in genome-wide association studies. *Nat Genet* **38**, 904-909, doi:10.1038/ng1847 (2006).

5 Tracy, C. A. & Widom, H. Level-spacing distributions and the Airy kernel. *Physics Letters B* **305**, 115-118 (1993).

6 Tracy, C. A. & Widom, H. Level-spacing distributions and the Airy kernel. *Communications in Mathematical Physics* **159**, 151-174 (1994).

7 Tracy, C. A. & Widom, H. Distribution functions for largest eigenvalues and their applications. *ICM 2012* **1**, 587-596 (2012).

8 Bradbury, P. J. *et al.* TASSEL: software for association mapping of complex traits in diverse samples. *Bioinformatics* **23**, 2633-2635, doi:10.1093/bioinformatics/btm308 (2007).

9 Griesbach, R. J. in *Flower breeding and genetics: Issues, challenges and opportunities for the 21st century* (ed N. O. Anderson) 301-336 (Springer, 2007).

10 Griesbach, R. J., Stehmann, J. R. & Meyer, F. Anthocyanins in the “red” flowers of Petunia exserta. *Phytochemistry* **51**, 525-528, doi:10.1016/S0031-9422(99)00026-6 (1999).

11 Ando, T. *et al.* Differences in the floral anthocyanin content of red petunias and Petunia exserta. *Phytochemistry* **54**, 495-501, doi:10.1016/S0031-9422(00)00113-8 (2000).

12 Kates, H. R., Johnson, M. G., Gardner, E. M., Zerega, N. J. C. & Wickett, N. J. Allele phasing has minimal impact on phylogenetic reconstruction from targeted nuclear gene sequences in a case study of Artocarpus. *Am J Bot* **105**, 404-416, doi:10.1002/ajb2.1068 (2018).

13 Lischer, H. E., Excoffier, L. & Heckel, G. Ignoring heterozygous sites biases phylogenomic estimates of divergence times: implications for the evolutionary history of microtus voles. *Mol Biol Evol* **31**, 817-831, doi:10.1093/molbev/mst271 (2014).

14 Zubko, E. *et al.* Activation tagging identifies a gene from Petunia hybrida responsible for the production of active cytokinins in plants. *Plant J* **29**, 797-808 (2002).

15 Napoli, C. A. & Ruehle, J. New Mutations Affecting Meristem Growth and Potential in   *Petunia hybrida*Vilm. *J. Hered.* **87**, 371-377 (1996).

16 Snowden, K. C. *et al.* The Decreased apical dominance1/Petunia hybrida CAROTENOID CLEAVAGE DIOXYGENASE8 gene affects branch production and plays a role in leaf senescence, root growth, and flower development. *Plant Cell* **17**, 746-759, doi:10.1105/tpc.104.027714 (2005).

17 Drummond, R. S. *et al.* Environmental control of branching in petunia. *Plant Physiol* **168**, 735-751, doi:10.1104/pp.15.00486 (2015).
